# Programmed Cell Death Protein 1/Programmed Cell Death Protein Ligand 1 Immunosuppressants in Advanced Non-Small Cell Lung Cancer Research Progress in Treatment

**DOI:** 10.3389/fphar.2022.918709

**Published:** 2022-06-16

**Authors:** Feng Li, Binchi Liao, Ting Wang, Tingting Qi, Yixin Wang

**Affiliations:** ^1^ Sichuan Cancer Hospital, Cancer Hospital Affiliate to School of Medicine, University of Electronic Science and Technology of China, Chengdu, China; ^2^ School of Medicine, University of Electronic Science and Technology of China, Chengdu, China; ^3^ National Medical Products Administration Institute of Executive Development, Beijing, China

**Keywords:** PD-1/PD-L1 inhibitors, NSCLC, clinical trials, adverse Reactions, immunity therapy

## Abstract

PD-1/PD-L1 play key roles in tumor immune escape and the formation of the tumor microenvironment, and are closely related to the generation and development of tumors. Blocking the PD-1/PD-L1 pathway can reshape the tumor microenvironment or block the formation of the tumor microenvironment and enhance endogenous antitumor immune response. Clinical trials show that the treatment of non-small cell lung cancer (NSCLC) with PD-1/PD-L1 inhibitors has significant advantages. The review briefly describes these basic principles of the PD-1/PD-L1 pathway and action mechanism in the treatment of NSCLC. A summary of global PD-1/PD-L1 clinical trials and five PD-1/PD-L1 inhibitors approved by FDA, EMA and NMPA for advanced NSCLC were analyzed.

## Introduction

The incidence and death rate of cancer around the world is increasing rapidly, and it has become a major “killer” affecting people’s survival and health. According to the statistics of the World Health Organization’s International Cancer Research Agency (Global Cancer Observatory, IARC), the number of cancer patients in 2020 will be 19.292789 million, and the death toll was 9,958,133 (IARC Global Cancer Observatory). Lung cancer is one of the most common malignant tumors in the world. Its incidence and mortality are second only to breast cancer. It is estimated that 1,796,144 people died of lung cancer, accounting for 18.0% of the total cancer deaths (Cao and Chen, 2021). It estimates that by 2030, the number of lung cancer patients worldwide may exceed 2.2 million, and 1.1 million patients may death (Arnold et al., 2017). The non-small cell lung cancer (NSCLC) accounts for about 80%–85% of the total number of lung cancers, and most NSCLC patients are already in advanced stage at the time of diagnosis (Rosell and Karachaliou, 2016). Chemotherapy has been the main traditional treatment for advanced NSCLC in the past few decades, but chemotherapy drugs not only attack tumor cells, but also damage healthy cells. Chemotherapy is usually accompanied by a series of serious adverse reactions, such as bone marrow suppression, digestive tract reactions, hair loss, etc. Gene testing is required before using the targeted drugs. The mutation of the target gene directly affects the efficacy of the targeted drug. However, there is currently no effective method to avoid disease progression caused by drug resistance. Recently, researches on immunity checkpoint inhibitors (ICIs) have progressed rapidly. Various new drugs have been developed in immunotherapy. The most significant clinical improvement is programmed cell death protein 1 (PD-1) and programmed cell death protein ligand 1 (PD-L1) monoclonal antibody (Li et al., 2018a). PD-1/PD-L1 inhibitors have produced significant survival benefits in the treatment of some advanced NSCLC, bringing hope to the treatment of all NSCLC patients.

A variety of PD-1/PD-L1 inhibitors have been approved for marketing. The PD-1 inhibitor includes Nivolumab, Pembrolizumab, Cemiplimab, Sintilimab, Toripalimab, Camrelizumab, Tislelizumab, etc. PD-L1 inhibitor includes Durvalumab, Atezolizumab, etc. There are 15 PD-1/PD-L1 inhibitors for NSCLC in clinical trials registered in clinicaltrials.gov until 15 January 2022 (Table 1). The some of the PD-1 inhibitors (i.e., Nivolumab, Pembrolizumab, Cemiplimab), and PD-L1 inhibitors (i.e., Durvalumab and Atezolizumab) have been authorized to market at home and abroad, and have shown higher survival benefits than chemotherapy in clinical trials or clinical applications. The PD-1/PD-L1 inhibitors, approved by the European Medicines Agency (EMA) and the United States Food and Drug Administration (FDA) and the China National Medical Products Administration (NMPA) for the treatment of advanced NSCLC (Xu et al., 2018) (Table 2) domestic preparations include Sintilimab (PD-1, February 2021), Camrelizumab (PD-1, December 2021), Tislelizumab (PD-1, June 2021; approved in China for the treatment of NSCLC). The review summarizes and analyzes the clinical studies of five currently used drugs targeting PD-1/PD-L1 immune checkpoints approved by FDA, EMA, and NMPA in advanced NSCLC.

**TABLE 1:** Summary of clinical trials of PD-1/PD-L1 immunosuppressants for NSCLC registered in clinicaltrials.gov.

S/N	Drug	Type	Manufacturer	Antibody Isotype	Global Clinical Trials	Completed	Chinese Clinical Trials	Approved
1	Pembrolizumab	PD-1	MSD	IgG4	103	12	15	US (2017), China (2018)
2	Nivolumab	PD-1	Bristol-Myers Squibb	IgG4	75	13	7	US (2014), China (2018)
3	Atezolizumab	PD-L1	Roche	IgG1	51	9	6	US (2016), China (2019)
4	Durvalumab	PD-L1	AstraZeneca	IgG1	51	2	7	US (2018), China (2019)
5	Sintilimab	PD-1	Innovent Biologics	IgG4	24	0	21	US(Not ),China (2020)
7	Camrelizumab	PD-1	Hengrui	IgG4	12	0	8	US(Not), China (Not)
8	Toripalimab	PD-1	Jun Real	IgG4	11	1	11	US (2021), China (2020)
9	Avelumab	PD-L1	Merck/Pfizer	IgG1	10	2	0	US (2017), China (Not)
10	Tislelizumab	PD-1	BeiGene	IgG4	10	0	8	US(Not), China (Not)
11	Cemiplimab	PD-1	Sanofi/Regeneron	IgG4	7	3	2	US (2021), China (2021)
12	Envafolimab	PD-L1	Corning Jerry	IgG1	2	0	2	US(Not), China (2020)
13	Sasanlimab	PD-1	Wuhan Costan	IgG4	2	1	1	US(Not), China (Not)
14	Dostarlimab	PD-1	TESARO/AnaptysBio	IgG4	2	0	0	US (2018), China (Not)
15	Sindelizumab	PD-1	Innovent Biologics/Eli Lilly	IgG4	1	0	1	US(Not), China (2018)

**TABLE 2:** FDA/EMA/NMPA-approved NSCLC treatments of PD-1/PD-L1 blocking antibodies.

Generic	Brand	Strength	Approved	Form	Adult Dose and Treatment Endpoints
Atezolizumab	Tecentriq	60 mg/ml	US EU China	soln for IV infusion after dilution	Single agent:840 mg/2weeks、1200 mg/3weeks、1680 mg/4 weeks; In combination with platinum-based chemotherapy: 1200 mg/3 weeks; after4–6 cycles of chemotherapy completed,and if bevacizumab discontinued, give 840 mg/2 weeks、1200 mg/3 weeks、1680 mg/4 weeks Continue until disease progression or unacceptable toxicity.
Cemiplimab	Libtayo	50 mg/ml	US EU	soln for IV infusion after dilution	350 mg/3 weeks until disease progression or unacceptable toxicity.
Durvalumab	Imfinzi	50 mg/ml	US EU China	soln for IV infusion after dilution	Stage III NSCLC(<30 kg):10 mg/kg/2 weeks;(≥30 kg): 10 mg/kg/2 weeks、1500 mg/4 weeks,Continue until disease progression, unacceptable toxicity.
Nivolumab	Opdivo	10 mg/ml	US EU China	soln for IV infusion after dilution	Single agent:240 mg/2 weeks或480 mg/4 weeks,until disease progression or unacceptable toxicity.
Pembrolizumab	Keytruda	25 mg/ml	US EU China	soln for IV infusion after dilution	Single agent:200 mg/3 weeks or 400 mg/6 weeks until disease progression or unacceptable toxicity.or up to 24 months in patients without disease progression.

## Mechanism of Programmed Cell Death Protein 1/Programmed Cell Death Protein Ligand 1 in Non-Small Cell Lung Cancer

**FIGURE 1:**
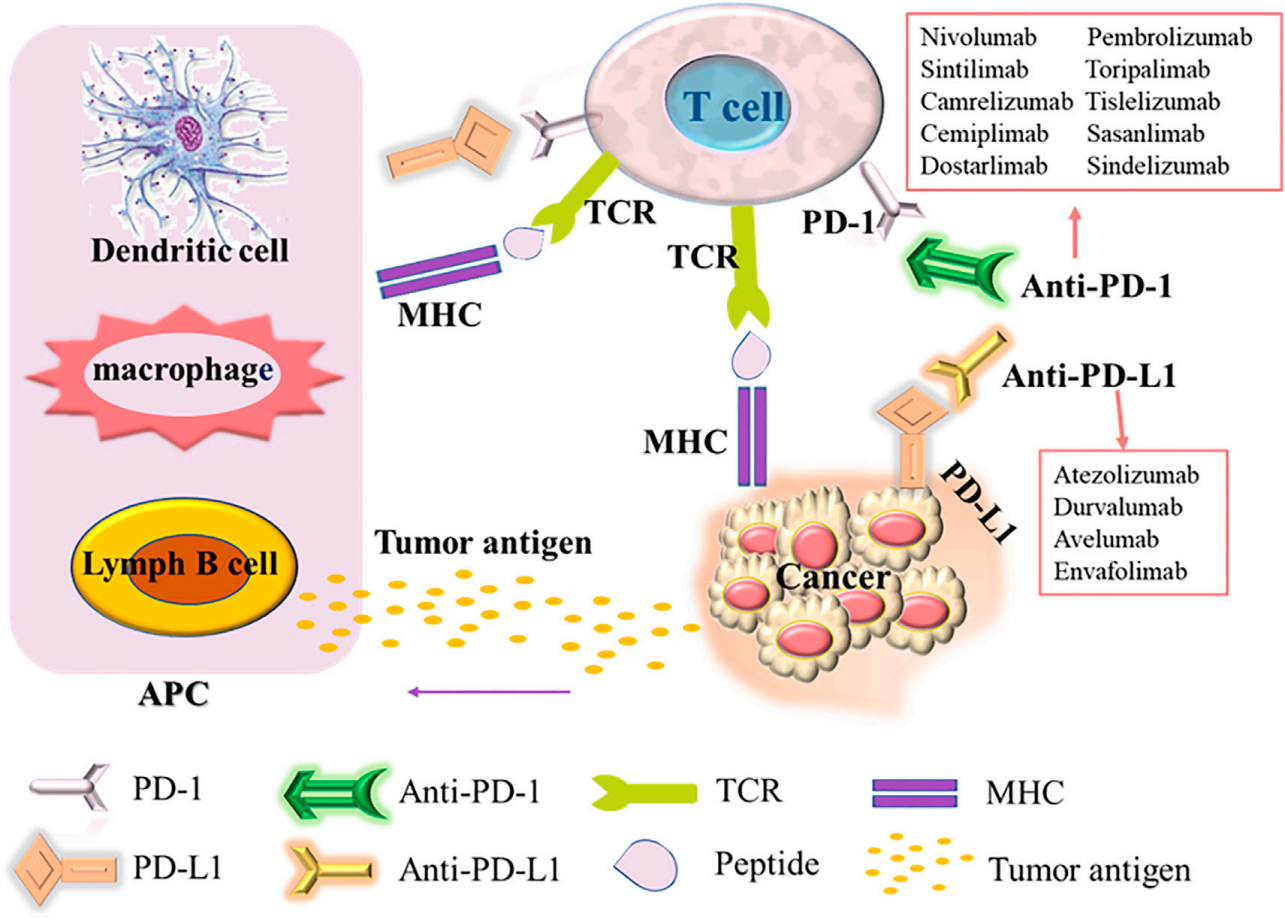
Mechanism of action of PD-1/PD-L1 inhibitors.

PD-1, also known as cluster of differentiation 279 (CD279), is a domain composed of 288 amino acid residues in the N-terminal of the immunoglobulin superfamily. It is an inhibitory protein receptor related to apoptosis. It is regarded as a sign of T cell unresponsiveness or exhaustion (Ishida et al., 1992). It is mainly expressed on the surface of T lymphocytes, B lymphocytes, dendritic cells (DC), NK cells (natural killer cells) and other cells (Calles et al., 2015), involved in autoimmune tolerance in a variety of physiological responses. As the major ligand of PD-1, PD-L1 (known as CD274 or B7-H1) is expressed in malignant tumor cells, lymphocytes, antigen presenting cells (APC), hematopoietic cells and epithelial cells. However, the expression of PD-L1 is usually low in stable state and upregulation induced by inflammatory stimulation (Yearley et al., 2017). In many cancers (including NSCLC), the PD-L1 signaling pathway is usually abnormally activated, and the interaction between PD-1 and PD-L1 inhibits the proliferation and activity of CD4+T cells and CD8+T cells, reducing their immune response to surrounding tissues and preventing the occurrence of autoimmune diseases (David et al., 2019; song et al., 2020). Thus in tumors, inflammation-induced PD-L1 expression in the tumor microenvironment causes PD-1-mediated T cell exhaustion, and suppresses anti-tumor cytotoxic T cell responses, leading to autoimmune killing of T cells in the tumor local microenvironment attenuated function. The tumor cells evade the immune system through various mechanisms, including low tumor cell immunogenicity, recognition of tumor-specific antibodies as self-antigens, tumor surface antigen modulation, tumor-induced privileged regions, tumor-induced immunosuppression, etc. Then, the tumor cells can avoid the immune system recognition and attack, achieve grow, metastasize and tumor immune escape, promoting tumor growth (Ribas, 2015; Ribas and Wolchok, 2018). Immune escape also occurs in the development of NSCLC. Tumor cells avoid the killing and elimination of the body’s autoimmunity, and establish a local microenvironment in some tissues that are conducive to tumor development. The mechanism of action of PD-1 and PD-L1 immunosuppressants is to block the PD-1 and PD-L1 signaling pathways of NSCLC cells (Figure 1), activate the immune activity of T cells, leading to the inhibition on the growth and proliferation of tumor cells, then achieving its final apoptosis (Li et al., 2018b).

Upon T cell activation, the PD-1 receptor binds to PD-L1expressed on the surface of cancer cells and suppresses the immune response. Antigen-presenting cells (APC) process antigens released by cancer cells and present them to T cells for promoting T cell activation and high PD-1 expression. In addition, tumor cells can also present antigens directly for activating T cells in the context of the major histocompatibility complex (MHC). PD-1/PD-L1 inhibitors can enhance the body’s immune response by blocking the binding of PD-1/PD-L1.

## Clinical Study of Programmed Cell Death Protein 1/Programmed Cell Death Protein Ligand 1 Immunosuppressant on Advanced Non-Small Cell Lung Cancer Treatment

Table 3 is a summary of the clinical trials with PD-1/PD-L1 inhibitors that are widely used in advanced NSCLC in China and other countries. The subsequent analysis and discussion are based on the clinical trials listed in Table 3.

**TABLE 3:** FDA/EMA/NMPA-approved NSCLC treatments of PD-1/PD-L1 blocking antibodies clinical trials.

Target	Drug	Study	PD-1/PD-L1 Group vs. Control Group	Inclusion Criteria	Study Type	Phase	N	OS( PD-1/PD-L1 Group vs. Control Group)
PD-1	Pembrolizumab	KEYN0TE-001 Garon et al. (2019)	Pembrolizumab/No Pembrolizumab	untreated advanced NSCLC	singlearm	Ⅰ	550	22.3/10.5
		KEYNOTE-010 Herbst et al. (2016)	Pembrolizumab/Docetaxel	Previously treated NSCLC with PD-1/PD-L1 expression >50%	RCT	Ⅱ/Ⅲ	1,034	12.7/8.5
		KEYNOTE-021 Langer et al. (2016)	Pembrolizumab + Pemetrexed + Carboplatin/Pemetrexed + Carboplatin	untreated advanced NSCLC	RCT	Ⅱ	123	21.4/16.4
		KEYNOTE-024 Brahmer et al. (2017)	Pembrolizumab/Carboplatin + pemetrexed or paclitaxel or cisplatin + gemcitabine	PD-1/PD-L1 expression>50%, no EGFR gene mutation	RCT	Ⅲ	305	30.0/14.2
		KEYNOTE-042 Mok et al. (2019)	Pembrolizumab/platinum-based + chemotherapy	No EGFR gene mutation or ALK translocation	RCT	Ⅲ	1,444	20.0/12.2
		KEYNOTE-189 Garassino et al. (2019)	Pembrolizumab + Chemo/chemo + placebo	NSCLC patients without metastasis and without EGFR gene mutation	RCT	Ⅱ/Ⅲ	616	20.2/13.5
		KEYNOTE-407 Paz-Ares et al. (2020)	Pembrolizumab + Chemo/chemo	Untreated, non-metastatic NSCLC	RCT	Ⅲ	559	15.9/11.3
	Nivoliumab	CheckMate 227 Hellmann et al. (2019)	Nivoliumab or Nivoliumab + ipilimumab/chemotherapy	Squamous/non-squamous stage IV or recurrent NSCLC	RCT	Ⅲ	1739	17.2/13.9
		CheckMate 9LA Paz-Ares et al. (2021)	Nivolumab + ipilimumab + chemotherapy/chemotherapy alone	Squamous/non-squamous stage IV or recurrent NSCLC	RCT	Ⅲ	719	14.1/10.7
		CheckMate 017 Horn et al. (2017)	Nivolumab/Docetaxel	Stage IIIB/IV squamous NSCLC	RCT	Ⅲ	272	9.2/6.0
		CheckMate 057 Borghaei et al. (2021)	Nivolumab/Docetaxel	Stage IIIB/IV non-squamous NSCLC	RCT	Ⅲ	582	^a^12.2/9.4
		NEOSTAR Cascone et al. (2021)	Nivolumab/Nivolumab + Ipilimumab	Operable NSCLC	RCT	Ⅱ	44	—
		CheckMate 078 Wu et al. (2019)	Nivolumab/Docetaxel	Stage IIIB, stage IV, recurrent squamous/non-squamous NSCLC after prior chemotherapy	RCT	Ⅲ	504	^a^12.3/7.9
		CheckMate 026 (Carbone et al. (2017)	Nivolumab/platinum chemotherapy	Squamous/non-squamous stage IV or recurrent NSCLC	RCT	Ⅲ	530	^a^14.4/13.2
	Cimepritimab	MA04.01 Moreno et al. (2018)	Cimepritimab/Cimepritimab + Chemotherapy	Advanced NSCLC	RCT	Ⅰ	53	—
		EMPOWER-lung1 Sezer et al. (2021)	Cimepritimab/platinum doublet chemotherapy	Stage IIIB/IV squamous/non-squamous NSCLC	RCT	Ⅲ	710	^a^22.1/14.3
		EMPOWER-Lung2 Rizvi et al. (2018)	Cimepritimab and ipilimumab ± chemotherapy/pembrolizumab	Advanced NSCLC with PD-L1≥50%	RCT	Ⅲ	585	—
		NCT03367819 Zucali et al. (2022)	Ixatuximab + Cimipritimab	Advanced NSCLC	singlearm	Ⅰ/Ⅱ	20	—
		MPOWER-Lung3^ ^ Gogishvili et al. (2021)	Cimepritimab + platinum-based doublet chemotherapy/platinum-based doublet chemotherapy	Metastatic NSCLC (Stage IV)/Locally Advanced NSCLC (Stage IIIB/C)	RCT	Ⅲ	466	22/13
		EMPOWER-Lung4 Shim et al. (2020)	Cimepritimab/Cimepritimab + Ipilimumab/Ipilimumab	Advanced NSCLC	RCT	Ⅱ	28	—
PD-L1	durvalumab	PACIFIC Faivre-Finn et al. (2021)	durvalumab/placebo	Unresectable Stage III NSCLC	RCT	Ⅲ	709	47.5/29.1
		NCT02904954 NasserAltorki et al. (2021)	durvalumab/durvalumab + radiotherapy	Resectable early stage NSCLC	RCT	Ⅱ	60	—
		ATLANTIC Garassino et al. (2018)	durvalumab	Advanced NSCLC progression after at least two prior systemic regimens	singlearm	Ⅱ	444	^a^13.6
		NCT02125461 Antonia et al. (2017)	durvalumab/placebo	Stage III, locally advanced, unresectable NSCLC	RCT	Ⅲ	713	^a^16.8/5.6
	Atezolizumab	IMpower131 Jotte et al. (2020)	Atezolizumab + chemotherapy/chemotherapy	Chemotherapy-naïve stage IV squamous NSCLC	RCT	Ⅲ	1,021	14.2/13.5
		POPLAR Fehrenbacher et al. (2016)	Atezolizumab/Docetaxel	NSCLC that has progressed after platinum-based chemotherapy	RCT	Ⅱ	287	12.6/9.7
		BIRCH Peters et al. (2017)	Atezolizumab	Stage IIIB/IV or recurrent NSCLC	singlearm	Ⅱ	659	^a^23.5/15.5/13.2
		IMpower110 Herbst et al. (2020)	Atezolizumab/chemotherapy	Stage IV non-squamous/squamous NSCLC	RCT	Ⅲ	572	^a^20.2/13.1
		OAK Fehrenbacher et al. (2018)	Atezolizumab/Docetaxel	Previously treated advanced NSCLC	RCT	Ⅲ	1,225	^a^13.8/9.6
		IMpower132 Nishio et al. (2021)	Atezolizumab + chemotherapy/chemotherapy	Primary chemotherapy for stage IV non-squamous NSCLC without sensitizing EGFR or ALK gene alterations	RCT	Ⅲ	578	^a^15.9/10.5
		IMpower150 Socinski et al. (2018)	Atezolizumab + chemotherapy/bevacizumab + chemotherapy	Chemotherapy-naive stage IV/recurrent metastatic non-squamous NSCLC	RCT	Ⅲ	356	^a^19.2/14.7

OS; overall survival, Time from randomization to death (from any cause).

a stands for Median Survival Time (mOS) .

RCT, randomized controlled trial.

### Pembrolizumab

Pembrolizumab monoclonal antibody (Keytruda; Merck, Sharp and Dohme Corp., Kenilworth, NJ) is a humanized monoclonal IgG4-κ isotype antibody. It blocks the interaction between PD-1 and PD-L1 by binding to the PD-1 receptor. In China 2018, the National Medical Products Administration (NMPA) officially approved Pembrolizumab on 28 March 2019 for: 1) a test approved by the National Medical Products Administration to evaluate the PD-L1 tumor proportion score (TPS) ≥1% epidermal growth factor receptor (EGFR) gene mutation-negative and anaplastic lymphoma kinase (ALK)-negative locally advanced or metastatic NSCLC first-line monotherapy, 2) combination with pemetrexed and platinum-based chemotherapy for the first-line treatment of epidermal growth factor receptor (EGFR) gene mutation-negative and anaplastic lymphoma kinase (ALK)-negative metastatic non-squamous NSCLC, 3) combination with carboplatin and paclitaxel for the first-line treatment of patients with metastatic squamous NSCLC. As of 15 January 2022, there are 372 clinical trials of pembrolizumab for NSCLC registered at clinicaltrials.gov, of which 35 are launched in China.

The efficacy of pembrolizumab monotherapy in patients with advanced NSCLC is showed in Table 3, including a phase I study (KEYNOTE-001) in previously treated and untreated disease, a second-line or higher setting Phase II/III study (KEYNOTE-010), and two Phase III studies in the first-line setting (KEYNOTE-024 and -042). In three randomized studies (KEYNOTE-010, -024, and -042), overall survival (OS) was significantly longer with pembrolizumab than with chemotherapy (Herbst et al., 2016; Brahmer et al., 2017; Garon et al., 2019; Mok et al., 2019). Clinical trials of pembrolizumab in combination with platinum-based chemotherapy showed improved efficacy compared with platinum-based chemotherapy alone, and in squamous NSCLC (KEYNOTE-407, phase III) and two non-squamous NSCLC clinical trials demonstrated the manageable safety profile (KEYNOTE-021 (Phase II), KEYNOTE-189 (Phase III)). In the KEYNOTE-021 cohort, the objective response rate (ORR) was 55% with pembrolizumab plus chemotherapy versus 29% with chemotherapy alone (Langer et al., 2016). Primary endpoints of OS and progression-free survival (PFS) both enhanced by the combination therapy in KEYNOTE-189 and KEYNOTE-407 (Garassino et al., 2019; Paz-Ares et al., 2020).

### Nivolumab

Nivolumab is a humanized monoclonal antibody (IgG4 subtype) directly against the programmed death 1 (PD-1) receptor, which is developed by Bristol-Myers Squibb. Until 15 January 2022, there are 255 clinical trials of nivolumab for NSCLC registered with clinicaltrials.gov, of which 22 are launched in China. Based on datasets from phase III randomized trials (CheckMate 017, 057) and phase II trials, FDA approved the National Comprehensive Cancer Network (NCCN) NSCLC panel recommend nivolumab as a treatment for metastatic squamous, metastatic preferred follow-up therapy for patients with non-squamous NSCLC (Table 3). On 15 June 2018, China approved it for the treatment of 1) epidermal growth factor receptor (EGFR) gene mutation-negative and anaplastic lymphoma kinase (ALK)-negative NSCLC; (2) previously received platinum-based chemotherapy Adult patients with locally advanced or metastatic NSCLC after disease progression or intolerance.

An OS benefit of Nivolumab over docetaxel was observed in patients with squamous NSCLC regardless of PD-1/PD-L1 expression levels. In patients with non-squamous NSCLC, higher levels of PD-L1 expression are associated with a greater OS benefit from Nivolumab, but treatment gains are also observed in patients with PD-1/PD-L1 expression <25%. Compared to docetaxel, the significant improvement in OS and tolerability of Nivolumab for NSCLC patients is progressed during or after platinum-based chemotherapy (Horn et al., 2017). The response of Nivolumab is durable and generally well-tolerated, with 14% of patients experiencing grade 3–4 treatment-related adverse events (Wu et al., 2019; Cascone et al., 2021), reflecting the unique advantage of the mechanism for the action of immunotherapy. In CheckMate 078, a Chinese-based Phase III clinical trial, compared with docetaxel in previously treated patients with advanced NSCLC, Nivolumab improves the OS. It is consistent with CheckMate 017 and 057 global trials (Horn et al., 2017; Borghaei et al., 2021).

### Cimiprizumab

Cimepritimab is an IgG4 monoclonal antibody against PD-1. In 2017, Cimiprizumab was approved for the treatment of metastatic cutaneous squamous cell carcinoma and locally advanced cutaneous squamous cell carcinoma, which is not suitable for surgery or radiation. It also has shown antitumor activity in other advanced solid tumors (Papadopoulos et al., 2020). The latest results from the phase III clinical trial EMPOWER-Lung1 show that compared with platinum-based doublet chemotherapy in patients with advanced NSCLC (stage IIIB/IIIC or IV) and high PD-L1 expression (≥50%), Cimepritimab treatment significantly improved overall survival and progression-free survival (Sezer et al., 2021). In 2021, Cimepritimab is approved for first-line treatment of patients with advanced NSCLC and PD-L1 expression ≥50% in the United States. Data from the trials collated for the review (Table 3) suggest that the combination therapy provides a survival benefit for patients regardless of the proportion of PD-L1 expression. From the perspective of protecting patients from chemotherapy toxicity, patients with a very high proportion of PD-L1, especially those greater than 90%, may be ideal candidates for Cimepritimab monotherapy. Compared with ICI plus chemotherapy, the most favorable risk-benefit ratio was obtained in patients with high PD-L1 expression.

### Durvalumab

Duvalumab is a selective, high-affinity human immunoglobulin G1 monoclonal antibody, and developed by AstraZeneca Pharmaceuticals LP. It can block the binding of PD-L1 to PD-1 and CD80, then enhance the anti-tumor T cell activity (Sezer et al., 2021). In 2017, the drug was approved by the United States Food and Drug Administration for the treatment of patients with unresectable, stage III NSCLC, who have not progressed after platinum-based chemotherapy and radiation therapy. In 2018, it was approved as consolidation immunotherapy for patients with stage III NSCLC after definitive chemoradiotherapy (CRT), and was the first drug to slow the progression of NSCLC (Aredo et al., 2021). In December 2019, it was approved for marketing in China. It is the first PD-L1 immunotherapy drug approved in China. Until 15 January 2022, there are 169 clinical trials on Nivolumab for NSCLC registered with clinicaltrials.gov, of which 19 are launched in China.

Recently, the study results from PACIFIC demonstrate that improved OS with durvalumab is widely observed regardless of PD-L1 expression (Papadopoulos et al., 2020). Compared with patients with low or no expression (PD-L1 expression <25%), the median progression-free survival was longer in EGFR−/ALK− NSCLC patients (PD-L1 expression ≥25%) (Garassino et al., 2018). A progression-free survival (PFS) benefit was also consistently observed with durvalumab in the NCT02125461 trial (Shim et al., 2020).

### Atezolizumab

Atezolizumab is a humanized immunoglobulin G1 monoclonal antibody against programmed death ligand 1 (PD-L1). In December 2019, the FDA approved Atezolizumab in combination with paclitaxel and carboplatin for the first-line treatment of patients with metastatic non-squamous NSCLC without EGFR and ALK mutations. Until 15 January 2022, there are 150 clinical trials of nivolumab for NSCLC registered with clinicaltrials.gov, of which 17 are launched in China (Part of the clinical trial data is shown in Table 3). The results of clinical trials IMpower-131, -132, -150, -110 showed that the overall survival of Atezolizumab in NSCLC patients with high PD-L1 expression was significantly longer than that of platinum-based chemotherapy (Herbst et al., 2020) (Nishio et al., 2021). In phase II POPLAR trial, Atezolizumab showed significant improvement compared with docetaxel in previously treated patients with advanced NSCLC (unselected for PD-L1 expression). The improvement in overall survival increased with increased PD-L1 expression, and patients with the lowest PD-L1 expression have similar overall survival of the docetaxel group (Fehrenbacher et al., 2016). Atezolizumab monotherapy was well tolerated in PD-L1-selected patients with advanced NSCLC in BIRCH trial (Peters et al., 2017). The status of PD-L1 expression serves as a predictive biomarker to identify patients, who most likely to benefit from Atezolizumab.

### Programmed Cell Death Protein 1/Programmed Cell Death Protein Ligand 1 Combined Clinical Trial

In the tumor microenvironment, PD-1 regulates T cell function. In lymph nodes, CTLA-4 inhibits the early activation and differentiation of T cells. Therefore, the combination of anti-PD-1/PD-L1 and anti-CTLA-4 is considered as a complementary therapy to trigger immune checkpoint inhibition. On the basis of the clinical trial of PD-1/PD-L1 in combination with it in Table 3 and clinical data from keynote-189, when Pembrolizumab combinate with platinum and pemetrexed as first-line therapy for metastatic non-squamous NSCLC, no change happens in EGFR or ALK. In the double-blind Phase Ⅲ Keyn-189 study, first-line Pembrolizumab plus Pemetrexel and platinum chemotherapy significantly improved OS (HR 0.49, *p* < 001), PFS (HR 0.52, *p* < 001), and ORR (47.6% vs. 18.9%, *p* < 001). In keynote-407, compared with placebo plus chemotherapy, Pembrolizumab plus carboplatin and paclitaxel/nab-paclitaxel (chemotherapy) significantly improved overall survival (OS) and progression-free survival (PFS) in previously untreated metastatic squamous NSCLC patients. According to the CheckMate9LA study, compared to chemotherapy alone, Nivolumab plus Ipilimumab combined with two cycles of chemotherapy shows a significantly improved survival benefit. In patients with TMB≥10 mutations/Mb, compared with nivolumab plus ipilimumab, there is a significant PFS advantage (increased probability and duration of response), according to published data from CheckMate227, Part 1. Nivolumab plus Ipilimumab produces a median OS of 23.0 months versus 16.7 months for chemotherapy, and an estimated relative reduction of 23% in the risk of death (HR: 0.77; 95%CI: 0.56–1.06). In the EMPOWER-Lung 3 study, Simiprizumab plus chemotherapy showed clinically and statistically significant improvements in OS, PFS, ORR, and DOR compared with chemotherapy alone, consistently in safety with Simiprizumab monotherapy and platinum-based chemotherapy. In the Global Open-label Phase III IMpower131 study, the addition of Atezolizumab to CnP provides PFS and OS benefits in patients with metastatic squamous NSCLC, whose tumors have high PD-L1 expression. In conclusion, compared to chemotherapy, PD-1/PD-L1 combination therapy significantly prolongs OS and PFS, and has a similar risk of grade 3–4 AE. It should be notice that PD-1/PD-L1 combination therapy increases the risk of AE discontinuation or death.

### Adverse Reactions of Programmed Cell Death Protein 1/Programmed Cell Death Protein Ligand 1 Inhibitors in Non-Small Cell Lung Cancer

The PD-1/PD-L1 axis acts as a brake on T cell activation and is involved in the pathogenesis of autoimmune diseases, including systemic lupus erythematosus (SLE) (Prokunina et al., 2002), rheumatoid arthritis (RA) (Wang et al., 2005), type I diabetes (TⅠD) (Curran and Sharon, 2017), autoimmune hepatitis (AIH) (Curran and Sharon, 2017), and multiple sclerosis(MS) (Kroner et al., 2005). Upregulation of PD-1/PD-L1 in the tumor microenvironment leads to impaired immune cell function and premature apoptosis, while autoimmune diseases are caused by excessive immunity, leading to the damage of normal tissues. The use of PD-1/PD-L1 inhibitors may cause dysregulation of the PD-1/PD-L1 signaling pathway, resulting in the loss of immune homeostasis and a strong abnormal immune response. The loss of immune homeostasis is related to genetic factors and environmental factors, and dietary factors or other diseases are also related. PD-1 receptors need to bind their ligands for suppressing the effects of immune responses. Therefore, the number of immune cells increases dramatically, and PD-1 expression on cells is affected by cytokine or Ag receptor stimulation. It could be the main reason for serious adverse reactions of PD-1/PD-L1 inhibitors during treatment.

Immunotherapy reactivates the immune system, and the body’s immune tolerance becomes unbalanced, leading to the emergence of new toxicity profiles known as immune-related adverse events (irAEs). These irAEs could affect multiple organ systems and tissues (Table 4), clinically manifest as autoimmune-like inflammatory side-effects, causing the damages on the skin, lungs, gastrointestinal tract, liver, endocrine glands, and skeletal muscle (Prokunina et al., 2002). Rash and pruritus are the most common cutaneous irAEs in NSCLC patients treated by anti-PD-1/PD-L1 immune checkpoint therapy. The other cutaneous tissue lesions include vitiligo, cutaneous capillary hyperplasia, lichenoid lichen, and bullous pemphigoid (Kong et al., 2005). Respiratory-related AEs also frequently occur with anti-PD-1/PD-L1 immunotherapy. Among them, immune-related pneumonia is common, including pulmonary sarcoidosis and tissue inflammatory pneumonia. The clinical symptoms are mainly dry cough, dyspnea, fever and Chest pain (Wang et al., 2005). The most common gastrointestinal toxicities during anti-PD-1/PD-L1 immunotherapy treatment are colitis and diarrhea, and the other gastrointestinal adverse reactions included decreased appetite, nausea, vomiting, and constipation (Curran and Sharon, 2017). In NSCLC patients receiving anti-PD-1/PD-L1 immunotherapy, the incidence of immune-related hepatitis is approximately 5%, whereas the incidence of severe hepatitis (grade III-IV) is <2%. The immune-mediated clinical symptoms of hepatitis include hepatomegaly, portal and periportal inflammation, lymphadenopathy, and infiltration of eosinophils, lymphocytes, and plasma cells (Kroner et al., 2005). In NSCLC patients, hypothyroidism is the most common endocrine toxicity (with an incidence of 5–15%). Hypophysitis, thyroiditis, hyperthyroidism, and adrenal insufficiency are also common immune-related endocrine diseases. The clinical symptoms are nonspecific heterosexual (common symptoms in cancer patients), such as fatigue, headache, and nausea (Naidoo et al., 2015).

**TABLE 4:** Major adverse events in PD-1/PD-L1 immunotherapy.

Human Body Systems/Tissues Etc.	Adverse Reaction Symptoms
Endocrine system	Hypothyroidism, Hyperthyroidism, Hypophysis, Thyroiditis, Adrenal insufficiency
Respiratory system	Pneumonia, Difficulty breathing
Cardiovascular system	Anemia, Thrombocytopenia, Neutropenia
Digestive system	Colitis, Diarrhea, Nausea, Constipation, Decreased appetite, Hepatitis, Alanine aminotransferase increased, Aspartate aminotransferase increased
Skin related	Rash, Pruritus, Vitiligo, Cutaneous capillary hyperplasia, Lichen lichenoides, Bullous pemphigoid
Musculoskeletal System	Myalgia, Joint pain
Other	Fatigue, Fever, Chills, Hair loss, Infusion reactions
Rarely (but serious)	Immune-related encephalitis, Myasthenia gravis, Acute renal failure, Interstitial nephritis, Myocarditis

## Discussion and Outlook

In this review, the clinical application progresses of the drug used in NSCLC was analyzed. It is found that the PD-1 inhibitors (e.g., Nivolumab, Cimipritimab and Bolizumab) and the PD-L1 inhibitors (e.g., Durvalumab and Atezolizumab) have benefited OS in clinical trials of NSCLC treatments. Although immune checkpoint inhibitors (ICIs) can improve the treatment landscape of NSCLC without EGFR, ALK or ROS1 aberrations, and reduce the proportion of chemotherapy in patients with high PD-L1 expression, leading to that these patients can avoid chemotherapy-related complications toxicity, the overall survival benefits still require to be improved by combination therapy (Huang et al., 2021). The degree of patient benefit is highly related to PD-1/PD-L1 pathway biomarkers. With the progresses of biomarker research, immunosuppressants will gain great achievements.

According to the relevant clinical trials analyzed in this review, PD-1/PD-L1 inhibitors can be considered as a class of drugs with relatively good safety, low toxicity and relatively good tolerance. The incidence and severity of immune-related adverse events (irAEs) varied widely across trials, including PD-1/PD-L1 inhibitor therapy-related adverse events. The incidence of adverse reactions, leading to the discontinuation of treatment, were significantly lower than those of docetaxel. It can be observed that PD-1/PD-L1 inhibitors are safe and can be tolerated by most patients. Combination therapy has a higher efficacy in NSCLC immunotherapy, e.g., the treatment by Ipilimumab plus Nivolumab is the first approved ICI combination therapy. However, the increased efficacy was also accompanied by higher frequency and more severe ADRs, with a greater probability of some clinically critical adverse events observed in combination therapy (Huang et al., 2021), such as diabetic ketoacidosis, thyrotoxic crisis, acute adrenal cortex insufficiency, myocarditis, non-infectious encephalitis/myelitis, Guillain-Barré syndrome, colitis, etc. Therefore, how to obtain higher efficacy and reduce or avoid serious adverse reactions in combination therapy is an important research direction in the future.

Prevention and reduction of the incidence on the adverse events can be obtained from: 1) monitoring of biomarkers can predict the occurrence of AEs during immunotherapy, such as serum thyroglobulin, thyroid autoantibodies and early changes in the levels of certain cytokines (IL-1β, increased levels of IL-2 and GM-CSF and decreased levels of IL-8, G-CSF, MCP-1), 2) comparing the changes of some biochemical indexes and imaging characteristics of tissues and organs before and after immunotherapy to assist the judgement on the possibility of irAEs (Wang et al., 2017). Routine baseline assessments include physical examination (height, weight, heart rate, blood pressure, and other general symptoms), imaging studies (chest CT, brain MRI), and laboratory tests (e.g., blood routine, blood chemistry, blood glucose, total bilirubin, TSH, free T4, LH, FSH, testosterone, cortisol, ACTH, infectious disease screening). In addition, tumor patients with different races, genders, and ages have different characteristics and severity of irAEs. Therefore, according to patients’ individual conditions, precise care is beneficial to reduce the incidence of AEs. For example, older adults with NSCLC often have comorbidities and polypharmacy, thus requiring adequate clinical monitoring.
